# Continuous ARterial monitoring in Elderly and Frail patients for hip fractUre surgery to prevent Low blood pressure – the CAREFUL Study Protocol

**DOI:** 10.1002/anr3.70059

**Published:** 2026-04-09

**Authors:** A. D. Kane, L. Kottam, J. Adamson, S. Davies, A. Mitchell, R. Sheridan, J. Dorey, S. Liggett, N. L. Clark, A. Lakhani, A. Rangan, G. Danjoux

**Affiliations:** ^1^ Department of Anaesthesia, James Cook University Hospital South Tees Hospitals NHS Foundation Trust Middlesbrough UK; ^2^ Hull York Medical School York UK; ^3^ North Yorkshire Academic Alliance of Peri‐operative Medicine; ^4^ Academic Centre for Surgery, South Tees Hospitals NHS Foundation Trust Middlesbrough UK; ^5^ York Trials Unit University of York York UK; ^6^ Department of Anaesthesia York and Scarborough Teaching Hospitals NHS Foundation Trust York UK; ^7^ Patient and Public Involvement representative; ^8^ Department of Orthopaedic Surgery, James Cook University Hospital South Tees Hospitals NHS Foundation Trust Middlesbrough UK

**Keywords:** arterial line, feasibility study, frailty, hip fracture, intra‐operative hypotension

## Abstract

Intra‐operative hypotension is common and associated with postoperative organ injury and mortality, particularly in older patients living with frailty. Continuous invasive arterial blood pressure monitoring provides real‐time measurements and may allow earlier detection and management of hypotension and improve outcomes. Contemporary UK data show that this intervention is rarely used in elderly patients living with frailty who are undergoing urgent hip fracture surgery. The CAREFUL Study (**C**ontinuous **AR**terial monitoring in **E**lderly and **F**rail patients for hip fract**U**re surgery to prevent **L**ow blood pressure) will synthesise evidence to determine the feasibility of a definitive randomised controlled trial evaluating continuous versus intermittent arterial blood pressure monitoring in this population. The study comprises three integrated workstreams: a systematic review and meta‐analysis to evaluate existing evidence on continuous arterial blood pressure monitoring and peri‐operative outcomes; mixed‐methods qualitative analysis to explore clinician attitudes, barriers and facilitators to implementation; a multi‐centre external feasibility randomised controlled trial with embedded qualitative research. The feasibility study will randomise 100 patients aged ≥ 65 years with a Clinical Frailty Scale score of ≥ 5 undergoing proximal femoral fracture surgery to either continuous invasive arterial blood pressure monitoring or non‐invasive 3‐ to 5‐minute cycle blood pressure monitoring. Data will include recruitment, retention and intervention compliance, exposure to intra‐operative hypotension, postoperative complications and quality of life. Findings will inform the design of a definitive effectiveness trial. Results will be shared through peer‐reviewed open‐access publications, stakeholder events and collaboration with professional bodies and patient partners to guide future peri‐operative care for older, frail surgical patients.

## Introduction

Intra‐operative hypotension occurs in up to two‐thirds of operations [1] and is associated with harm, particularly in high‐risk patient groups [2]. Large systematic reviews and meta‐analyses show that the severity of hypotension, both in terms of depth and duration, is linked to renal, cerebral and myocardial injury and mortality [2]. Patients at high risk of harm include those who are older (≥ 65 years) and living with frailty [3, 4]. This older and frail group represents one in four patients over 65 years presenting for surgery in the United Kingdom, rising to over 60% of those aged > 85 years [5], and will likely increase in number in the future [6, 7]. Notably, patients requiring surgery for proximal femoral fracture (PFF) are firmly in this demographic. Of over 70,000 patients with PFF having surgical treatment annually in England, Wales and Northern Ireland, 30‐day mortality is 6% and 1‐year mortality is up to 30% [8, 9].

In the United Kingdom, the minimum acceptable monitoring standard during anaesthesia includes intermittent non‐invasive blood pressure monitoring (typically every 3–5 minutes). National guidelines advise that additional invasive monitoring should be considered when there is cardiovascular comorbidity, frailty or the surgery is urgent [10]. However, recent data from the Royal College of Anaesthetists (RCoA) 7th National Audit Project (NAP7) on peri‐operative cardiac arrest highlights that this is not occurring in older frail patients, particularly those presenting for PFF surgery [5, 11, 12]. The report concluded that patient harm, including deaths, may have been prevented with increased rates of continuous invasive arterial blood pressure monitoring [5, 6, 12, 13, 14]. A key recommendation was that further research should evaluate if continuous blood pressure monitoring, as an intervention, is beneficial in this population [12].

Here, we aim to combine three complementary work streams to generate evidence, asking ‘*Does continuous arterial blood pressure monitoring during surgery benefit patients who are high risk, particularly those who are older and living with frailty?*. We will:

1. Undertake a systematic review and meta‐analysis to examine the current evidence base regarding the impact of continuous blood pressure monitoring on intra‐operative hypotension and associated morbidity and mortality.
2. Understand clinician attitudes towards continuous arterial monitoring in older frail patients presenting for trauma surgery through mixed‐methods qualitative research.
3. Undertake a feasibility study with embedded qualitative research to assess whether a full‐scale definitive randomised controlled trial (RCT) of continuous invasive arterial blood pressure monitoring versus standard care is feasible in the older, frail patient population presenting for hip fracture surgery.

## Methods

### Work package 1: systematic review

The systematic review will ask: ‘*Does continuous arterial blood pressure monitoring prevent exposure to intra‐operative hypotension and reduce morbidity and mortality?*’ and has been prospectively registered with PROSPERO (CRD42025648753). The review will be reported to PRISMA (Preferred Reporting Items for Systematic reviews and Meta‐Analyses) standards [15].

We will search databases including MEDLINE, EMBASE, PubMed, ClinicalTrials.gov and the WHO International Clinical Trials Registry Platform for studies reported in English. Observational data and RCTs will be included. Studies will also be identified through reference and citation tracking of included articles. Study authors may be contacted to request further information. The following search terms will be used: [((intra‐arterial) OR (continuous blood pressure) OR (invasive blood pressure) OR (arterial cannulation) OR (clearsight) OR (nexfin) OR (continuous non‐invasive) OR (continuous non‐invasive) OR (CNAP)) AND ((intra‐operative hypotension) OR (intraoperative hypotension) OR (hypotension)) AND ((anesthesia) OR (anaesthesia) OR (surgery))].

The review will include RCTs and observational data, such as cohort studies, but will exclude case studies and case series. Participants will be adult patients (≥ 18 years) undergoing a surgical procedure under the care of an anaesthetist and receiving any form of general anaesthesia and/or neuraxial anaesthesia (spinal, epidural or combined spinal‐epidural). Studies will be excluded if they focus solely on children (≤ 17 years), anaesthesia or sedation which is not intended to enable surgery, patients who are already anaesthetised before transfer to the operating theatre complex, anaesthesia not administered by anaesthetists, sedation only and non‐human studies.

The intervention to be examined will be the use of continuous arterial blood pressure monitoring in adult patients undergoing surgery using either non‐invasive or invasive monitoring and will be compared to standard care, namely intra‐operative blood pressure monitoring with non‐invasive intermittent (typically 3‐ to 5‐minute cycles) oscillometric blood pressure monitoring. Outcomes will include exposure to intra‐operative hypotension (time weighted average of mean arterial blood pressure less than 65 mmHg); morbidity, including rates of acute kidney injury, myocardial injury and delirium; and mortality. The outcomes are detailed in Supporting Information 1.

The review will be managed using Covidence (Covidence systematic review software, Veritas Health Innovation, Melbourne, Australia. Available at www.covidence.org). Following de‐duplication, titles and abstracts, followed by remaining full‐text articles, will be screened against the inclusion criteria by two reviewers, with a third assessor to resolve disagreements. All efforts will be made to retrieve the full‐text articles of potentially eligible articles.

Data extraction will occur independently by two reviewers in duplicate. A PRISMA flow diagram will illustrate the process.

Key study, participant and quality measures will be presented. Data from RCTs will be pooled in a random‐effects meta‐analysis, provided there are sufficient RCTs. Data for each measure will be presented as pooled odds ratios, mean differences or standardised mean differences, along with the associated 95% confidence intervals. A narrative synthesis of study results will be performed if substantial clinical or methodological heterogeneity is observed. A meta‐analysis may not be performed if substantial clinical or methodological heterogeneity is observed. All studies included in the analysis will be reviewed according to the Cochrane risk‐of‐bias tool 2 for randomised trials [16].

### Work package 2: mixed‐methods qualitative study

An online survey to determine current practices and attitudes of practicing anaesthetists towards continuous blood pressure monitoring in this patient group, with qualitative interviews.

We will aim to explore:

- respondent's experience in anaesthesia;
- experience of managing older frail patients;
- individual thresholds for arterial monitoring;
- perceived benefits, risks and harms of arterial monitoring;
- views on key outcome measures for a definitive study;
- equipoise and willingness to participate in a RCT.

We aim to survey anaesthetists of all grades (consultants, doctors in training and non‐consultant grades) who are currently practicing in the United Kingdom. National distribution networks (resident doctor networks, RCoA and the Association of Anaesthetists) will be utilised to ensure a large, representative sample of active anaesthetists based on previous experience.

Three focus groups (comprising 3–7 participants per group, approximately 15 participants in total) will be conducted. Key stakeholders (anaesthetists, orthopaedic surgeons, orthogeriatric physicians, operating department practitioners, representatives of the RCoA and Centre for Peri‐operative Care) will discuss clinical decision‐making regarding intra‐operative blood pressure monitoring and ascertain facilitators and barriers to running a full‐scale trial and the potential of changing practice.

The focus groups will explore the capacity and willingness to participate in a trial in the context of other ongoing trials in anaesthesia and the scope of inclusion criteria. We are interested in the potential reasons that prevent the wide‐scale implementation of continuous monitoring before and after trial evidence is available, as well as implementation issues. We will ascertain professional stakeholder views on aspects of trial design, including eligibility criteria and outcome measures.

### Work package 3: feasibility study

A feasibility study consisting of an external, multi‐centre, two‐arm, parallel‐group, assessor‐blinded randomised pilot trial with a nested qualitative study will be undertaken to determine the:

- feasibility of patient recruitment, intervention delivery, crossover rate, retention rates and completion of data collection;
- acceptability of the intervention to patients and carers;
- optimum primary outcome measure;
- feasibility of collecting health economic measures to inform the cost‐effectiveness evaluation in the main trial;
- feasibility of blinding outcome assessors to trial allocation.

### Setting

The study will be set in up to four National Health Service (NHS) hospitals in the UK providing anaesthesia and surgery for proximal femoral fracture.

### Inclusion criteria

Patients who meet all the following criteria will be considered eligible for the study:

- ≥ 65 years old;
- clinically frail – assessed as a Clinical Frailty Scale (CFS) Score of ≥ 5 [3];
- primary hip (proximal femoral) fracture.

### Exclusion criteria

Patients who meet any of the following criteria will not be considered eligible for the study:

- planned continuous arterial blood pressure monitoring or use of non‐invasive blood pressure monitoring at a high frequency (1‐minute cycles) throughout the case;
- declining consent.

### Intervention and comparator

Following the randomisation procedure below, participants will be allocated to either:

*(A) Continuous invasive arterial blood pressure monitoring*

- Up to three attempts to establish continuous invasive arterial blood pressure monitoring using local anaesthesia before general, neuraxial or regional anaesthesia.
- Continuous arterial blood pressure monitoring will be maintained until the patient reaches the recovery room, where it may be discontinued or continued at the discretion of the clinical team.

Or

*(B) Standard care*

- Three‐ to five‐minute cycles of non‐invasive blood pressure monitoring.

### Timeline and outcomes

**Figure 1:**
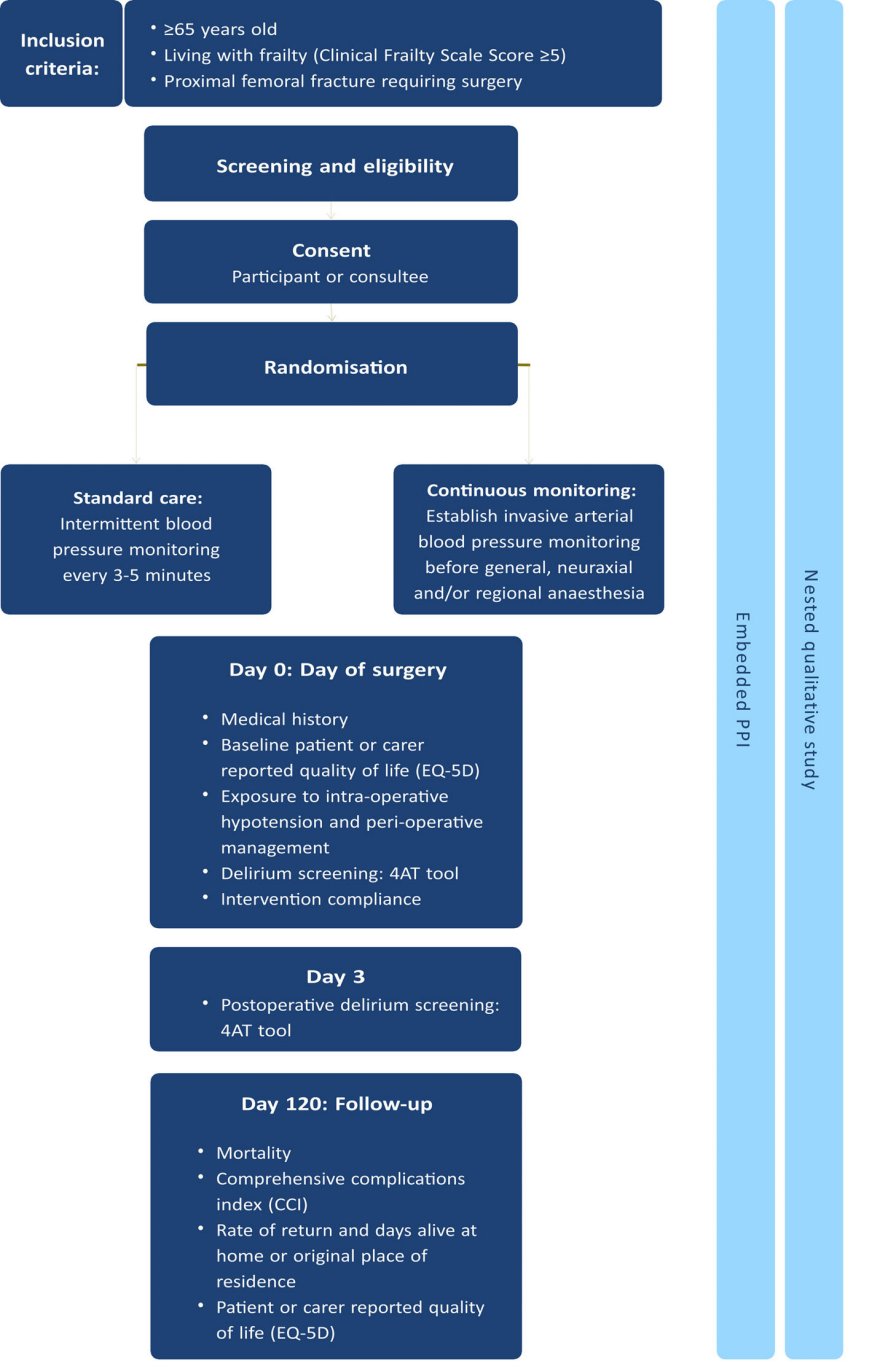
Overview of the external multi‐centre randomised feasibility trial – The CAREFUL study.

The main outcome is to assess feasibility. All data fields to be collected are described in Supporting Information 2 and the key study timepoints are shown in Fig. 1. Given the emergency setting of the study, eligibility, consent and randomisation may all occur on the day of surgery (day 0). A structured medical history will be recorded at the point of consent, and patient‐ or carer‐reported outcome measures (EQ‐5D) will be collected from pre‐fracture status. Delirium screening using the 4AT tool will occur before surgery and on day 3 (±1). Intra‐operative management and blood pressure will be recorded along with intervention compliance on the day of surgery. At 120 days, we will record mortality, the occurrence of major complications, days alive and at home in the original place of residence and patient‐ or carer‐reported outcomes (EQ‐5D).

### Harms

As major complications occur at high rates in this population, they will be collected and reported as clinical outcomes complications in all cases (see Supporting Information 2). If a local investigator considers that harm may be due to the intervention, an adverse event (AE) or serious adverse event (SAE) will be reported (see Supporting Information 3). This approach is in line with other interventional studies in peri‐operative medicine where high rates of significant complications are expected within the population of interest [17].

### Sample size

Sample sizes of up to 70 participants have been recommended for pilot trials to estimate the standard deviation of a continuous outcome for use in the power calculation of a full‐scale trial [18]. We will randomise 100 participants to allow for an attrition rate of 30% to achieve a minimum of 70 analysable participants at follow‐up.

### Participant selection and enrolment

All patients presenting for orthopaedic trauma surgery with proximal hip fractures at participating sites will be screened, and a log will be maintained on REDCap. Eligibility will be confirmed by a delegated member of the research team and recorded on REDCap.

The CAREFUL Study will employ a three‐layer consent process (patient consent, personal consultee approach and clinical consultee) designed to maximise inclusivity enabling as many patients as possible to participate, including those who lack capacity to consent and potentially benefit from the study (Supporting Information 4, S4).

Participation ends once the participant has completed the 120‐day follow‐up and all other data have been finalised. Participants are free to withdraw from the study following screening and consent, up until the final data collection point.

### Randomisation

Participants will be stratified by site, formed of randomly permuted blocks of randomly varying size using a 1:1 allocation ratio. The allocation sequence will be generated by a statistician at York Trials Unit and not revealed to the study team. The sequences for each site will be uploaded to the REDCap platform. Randomisation will be performed following consent and clinical teams will be informed as early as possible to allow planning of care. Randomisation will only be possible once the consent process is marked as complete on REDCap.

As this feasibility study is testing a pragmatic clinical trial design, clinical teams and patients will know the intervention allocation. Outcome assessors will be blinded to allocation.

### Data collection

All data will be collected electronically and captured in REDCap as per the timeline and Case Record Fields (see Fig. 1, Supporting Information 2). Details of data entry, storage and information governance are provided in Supporting Information 5.

### Statistical analysis plan

Full analyses will be detailed in a statistical analysis plan (SAP), which will be finalised before the end of data collection and reviewed and approved by the trial steering committee. A CONSORT diagram will report the flow of participants through the study. The recruitment rate and 95% confidence interval will be estimated. Outcomes and their completeness will be summarised descriptively by randomised group: continuous data using the mean, standard deviation, median and 25th and 75th percentiles; categorical data using the number of events and percentages. Standard deviations of continuous outcome measures at follow‐up time points will be estimated alongside 80% confidence intervals [18]. Statistical hypothesis testing of effectiveness will not be carried out. Treatment crossovers will be reported with reasons. Instances of unblinding of outcome assessors will be reported descriptively by randomised group.

### Nested qualitative study

To complement the focus groups in work package 2, we will collect additional qualitative information throughout the feasibility study setup and the duration of the study itself.

Data collection will take several forms, including document review of data captured during study setup and conduct and individual interviews:

- Minutes from trial management group meetings to keep abreast of any challenges that occur during the setup period of the trial, in particular matters arising regarding the recruitment of the four feasibility study sites. The team will then develop strategies to address the identified issues and incorporate this information into the full‐scale trial design.
- Expression of interest forms regarding site recruitment forms will be reviewed and tailored to capture training needs and any concerns they may have around participating and in the case of those who decline, an option to explain why they have chosen not to participate.
- Record and attend site initiation visits to note the main concerns sites express.
- For those sites recruited, verbal feedback will be sought to identify early challenges. The trial team will work with the sites to identify solutions to the issues raised. These solutions will be incorporated into the setup of future sites and full‐scale trial design.
- Semi‐structured interviews with clinical stakeholders from each pilot trial site (including anaesthetists, surgeons, operating department practitioners and research nurses; up to 16 – approximately four from each site). Data collection will focus on the views and experiences of delivering the intervention and recruiting participants for the trial.
- Semi‐structured interviews will be conducted with patients or their carers who participate (approximately 10) and decline participation (approximately 5) in the feasibility trial. We will ascertain their views on the study, the intervention, the trial processes and the outcomes which are important to them. Participants will be purposely selected (based on socio‐demographic characteristics, frailty and site) and interviewed following the final outcome measure collection. We are committed to recruiting under‐served populations into the qualitative study. Topic guides will be co‐produced with patient and public members.

Focus group and data from the nested qualitative study will be transcribed. All transcripts and documents collected will be thematically assessed, focusing on the feasibility and design of a full‐scale trial.

In addition, we will use the Implementation Outcomes Framework, focusing on the constructs of acceptability, fidelity, feasibility, adoption, appropriateness and sustainability to enhance our understanding of how to optimise the implementation of the intervention accounting for contextual factors and intended and unintended consequences of doing so, from a staff and patient perspective [19].

### Study monitoring and registration

Study monitoring procedure and sponsorship are reported in Supporting Information 6. The feasibility study has been prospectively registered with the ISRCTN (International Standard Randomised Controlled Trial Number) UK Clinical Study Registry (https://doi.org/10.1186/ISRCTN12558538). The reporting of this protocol is in line with SPIRIT (Standard Protocol Items: Recommendations for Interventional Trials) and the checklist is provided (Supporting information 7).

### Progression criteria

The feasibility study is not powered to detect any difference in clinical or patient‐focused endpoints. Therefore, progression criteria will centre on the deliverability of a definitive trial through evidence obtained in all three work packages. Progression criteria include:

- Work package 1: The systematic review supports that there is a lack of evidence in this area.
- Work package 2: Survey and qualitative interviews do not raise significant concerns about acceptability of the intervention to patients and carers, and clinical equipoise or barriers to large‐scale intervention implementation.
- Work package 3: We will use a red‐amber‐green rating system to classify key elements of feasibility (see Table 1). These will be:
  ○ Site setup: Four sites setup and recruiting patients
  ○ Recruitment: An average of two participants per site recruited per month, ensuring planned recruitment within 15 months.
  ○ Data collection: We anticipate a complete capture of patient‐reported outcome measures (EQ‐5D) at 120 days from 70% or more patients or their proxy.
  ○ Intervention compliance: High rates of intervention compliance and delivery (> 80%).
- If all scores are green, this will support progression towards a full trial. Amber scores will indicate that some modifications may be required for a full trial. Any red score will signal concern over feasibility. If none of the scores are green, combined learning from the nested qualitative study and work packages 1 and 2 may indicate how to overcome aspects of feasibility, which may provide reassurance for progression.

**Table 1:** Indicative progression criteria for feasibility. These criteria will be considered alongside findings from the systematic review and the mixed‐methods study to inform progression to a definitive randomised controlled trial.

Domain	Target at end of trial	Green	Amber	Red
Site setup	Four sites setup and recruiting first participant	100% (4 sites)	50–99% (2–3 sites)	< 50% (1 site)
Participant recruitment	Average of 2 participants recruited per site per month	100% (2 per month)	50–99% (1.0–1.9 per month)	< 50% (< 1 per month)
Outcome follow‐up data	Collect ≥ 70% of participant‐reported outcome data at 120 days	≥ 70% (≥ 70 patients)	50–69% (50–69 patients)	< 50% (< 50 patients)
Intervention compliance	≥ 80% of participants receive allocated treatment	≥ 80% (≥ 80 patients)	50–79% (50–79 patients)	< 50% (< 50 patients)

### Patient and public involvement (PPI) and engagement

A dedicated patient advisory group (PAG) of six to eight committed PPI members with lived experiences will be formed. If needed, PPI members will be signposted to various resources and provided with one‐to‐one training. Most activities will be virtual, enabling participation regardless of geographical location. Patient and public members' time and contributions are fully costed using the National Institute for Health and Care Research (NIHR) guidelines. The patient advisory group and PPI representatives will co‐develop all patient‐facing trial documentation, provide input into the design of qualitative interview guides, advise on matters arising and contribute to the final report.

Patient and public involvement will enable dissemination by supporting the development of leaflets, videos and input to professional societies and groups, as appropriate. The Guidance for Reporting Involvement of Patients and the Public (GRIPP2) reporting checklist will be used to publish the contribution and impact of PPI during the study and will be included in the final study report. An impact log will be maintained to capture and evaluate the overall impact of PPI based on the Public Involvement Impact Assessment Framework.

A stakeholder event at the end of the project as part of our assessment of progression, where PPI and other stakeholders will be fully embedded and central to the outcome. Our public dissemination strategy will be co‐developed with the patient advisory group and they will help with dissemination activities by supporting the development of results leaflets or content for various platforms, including websites, social media, news articles, professional societies and groups and patient charities.

### Dissemination plans

A dissemination and publication policy will be developed with an agreement between the trial management group, including ownership and publication rights. The policy and agreement will ensure that the publication process is organised fairly, balanced and transparently. The trial management group will be responsible for overseeing these arrangements.

We will host a stakeholder event, including PPI representation, to disseminate the findings, gather feedback and guide the development of a definitive study. We will communicate the results to groups including Royal Colleges and the Centre for Peri‐operative Care to inform future iterations of peri‐operative guidelines for patients living with frailty.

Findings will be presented at conferences and meetings, and feedback will be given to the PPI group.

## Discussion

Intra‐operative hypotension is common [1], associated with harm [2] and appears more severe in older patients living with frailty [4]. Emerging evidence supports that exposure to intra‐operative hypotension may be mitigated by establishing continuous arterial blood pressure monitoring before induction of anaesthesia, be it invasive [20] or continuous non‐invasive [21] for elective major surgery; however, this has not been evaluated in the emergency setting, nor have long‐term clinical or patient‐reported outcome measures been assessed for this intervention.

Recommendations from NAP7, which focused on peri‐operative cardiac arrest, supported a lower threshold for the use of continuous arterial blood pressure monitoring in high‐risk cases, but also acknowledged the lack of an evidence base for this intervention [12]. This was driven by both an observation that multiple objective high‐risk cases progressed to cardiac arrest without the use of continuous blood pressure monitoring [5] and that use of continuous monitoring was low in some high‐risk patient groups [11]. For example, older frail orthopaedic trauma patients had continuous invasive blood pressure monitoring in only 7% of cases at the start of their anaesthetic, and only 12% had continuous monitoring at any point [11, 12]. This is in contrast to other high‐risk groups where rates of use are considerably higher (cardiac surgery 80%, neurosurgery 50%) [12]. Even when adjusted for their estimated SORT score (Surgical Outcomes Risk Tool), those who were objectively high‐risk (5% or greater 30‐day predicted mortality) had only a 16% chance of continuous arterial blood pressure monitoring versus almost all other patient groups where a similar risk profile led to over 50% of cases having arterial monitoring [11]. This stark fact highlights the inequality that older trauma patients living with frailty may be missing the opportunity for patient benefit through what is a cheap and low‐risk intervention.

In adult patients undergoing major non‐cardiac surgery, data support that providing continuous blood pressure measurements detects about twice the number of intra‐operative hypotensive episodes versus intermittent non‐invasive monitoring [22]. Further, recent RCT data support that if invasive arterial monitoring is established before the induction of anaesthesia, then the area under a mean arterial pressure of 65 mmHg was reduced from 46 to 15 mmHg effectively reducing the exposure or ‘dose’ of hypotension by 66% [20]. In these studies, no target blood pressure value has been set; inserting an invasive blood pressure monitor was the patient‐protective intervention. Hypotension was managed as per the clinician's choice. Other patient benefits include easy blood gas analysis and sampling, which aid clinical decision‐making. Few studies have investigated if preventing peri‐operative hypotension reduces harm [23, 24]. However, there are suggestions that maintaining blood pressure within 10% of a patient's usual blood pressure reduces the risk of postoperative organ dysfunction as a composite measure [25].

Whilst NAP7 recommended higher rates of continuous monitoring, no study has investigated whether continuous arterial blood pressure monitoring can prevent hypotension and end‐organ injury, or improve the quality of life of older patients living with frailty who require urgent surgery. The CAREFUL study will determine if a definitive trial to address this question is feasible.

## Supporting information


**Supporting Information 1.** Systematic review data fields.

**Supporting Information 2.** Case record form fields.

**Supporting Information 3.** Adverse and serious adverse events.

**Supporting Information 4.** Consent details.

**Supporting Information 5.** Data management and storage.

**Supporting Information 6.** Study monitoring and sponsorship.

**Supporting Information 7.** SPIRIT checklist (Standard Protocol Items: Recommendations for Interventional Trials).
