# Supplementary material for: Continuous ARterial monitoring in Elderly and Frail patients for hip fractUre surgery to prevent Low blood pressure – the CAREFUL Study Protocol
Source: Anaesth Rep. 2026 Apr 9;14(1):e70059. doi: 10.1002/anr3.70059 (PMC13062759; doi:10.1002/anr3.70059)
Supplement: Supplementary file 1 — Supporting Information 1. Systematic review data fields. [file ANR3-14-e70059-s003.docx]

**Supporting information 1: Systematic review data fields**

**Main outcomes**

Time weighted average (TWA) of mean arterial pressure (MAP) less than that defined as primary endpoint of study.

**Additional outcomes**

TWA-MAP <75 mmHg

TWA-MAP <70 mmHg

TWA-MAP <65 mmHg

TWA-MAP <60 mmHg

TWA-MAP <55 mmHg

TWA-MAP <50 mmHg

Number of hypotensive episodes

Total duration of hypotensive episodes

Rate of diagnosis of acute kidney injury

Rise in creatinine

Rate of diagnosis of myocardial injury

Rise in troponin

Rate of postoperative delirium

Mortality rate

Fluid administered during the case

Vasopressor administration during the case
