# Supplementary material for: Continuous ARterial monitoring in Elderly and Frail patients for hip fractUre surgery to prevent Low blood pressure – the CAREFUL Study Protocol
Source: Anaesth Rep. 2026 Apr 9;14(1):e70059. doi: 10.1002/anr3.70059 (PMC13062759; doi:10.1002/anr3.70059)
Supplement: Supplementary file 2 — Supporting Information 2. Case record form fields. [file ANR3-14-e70059-s007.docx]

**Supporting information 2: Case record form fields**

The following data will be collected:

- **Recruitment rate:**
  - The number of screened and eligible patients.
  - Patients approached.
  - Number who provide consent.
  - Number of patients consented and randomised.
- **Treatment crossover, retention and unblinding.** The proportion of patients randomised who do not receive the allocated treatment,* the proportion dropping out between randomisation and follow-up, and arterial line failure rate. Number of outcome assessors unblinded to trial allocation.
- **Pre-operative data including:**
  - NHS number.
  - Home address, including postcode.
  - Height and weight.
  - Sex.
  - Time and date of presentation to hospital.
  - Type and site of hip fracture.
  - Patient or carer reported pre-injury mobility.
  - Pre-injury patient (or consultee) reported quality of life (EuroQol EQ5D5L questionnaire).
  - Pre-injury residential status.
  - Delirium screening (4AT tool).
  - Do not attempt cardiopulmonary resuscitation (DNACPR) status.
  - Comorbidities.
  - Drug history.
  - Routine blood test results on admission (routine blood test results on admission, if performed: full blood count, urea and electrolytes, clotting screen, CRP).
  - Blood pressure on admission.
- **Intra-operative data (all should be recorded on most standard anaesthetic charts):**
  - Peri-operative time stamps (into the anaesthetic room, into theatre, knife to skin, into recovery).
  - Grade of the most senior anaesthetist.
  - ASA physical status, as recorded on the chart.
  - Type of anaesthesia.
  - Total volume of crystalloid and colloid fluid administered in theatre.
  - Units of blood products administered in theatre.
  - Vasopressor and inotrope use in theatre.
  - Estimated blood loss.
  - Cardiac arrest in theatre.
  - Death in theatre.
  - Upload of intra-operative blood pressure measurements (see ‘Quantification of intra-opeartive hypotension’ below).
  - First blood pressure in recovery.
  - Immediate postoperative destination (ward, post-anaesthesia care unit, high dependency or critical care).
- **Quantification of intra-operative hypotension**
  - We will work with sites to collect intra-operative blood pressure data. Where charting is electronic, the preference would be a time-stamped record of systolic, mean and diastolic blood pressure with labels indicating the time into the anaesthetic room, time into theatre, knife to skin, end of surgery and time into recovery. Where this is not possible, a high-resolution image of the summary of the electronic chart would be acceptable. For sites which record data on paper charts, a high-resolution, de-identified scanned copy of the chart can be uploaded as a PDF or JPEG to REDCap, or a digital export from a monitor, or a digital image capture of blood pressures. Specific measures of hypotension will be assessed centrally by the study team. They will include: time-weighted average of mean arterial pressure (MAP) under 65 mmHg in the first 15 and 30 min of surgery and through the total duration of time in theatre, the number of discrete hypotensive episodes (MAP <65 mmHg), total hypotensive time (sum of all time where MAP <65 mmHg).
- **Intervention details:**
  - Group allocation (intervention or standard care).
  - Intervention compliance and crossover and reasons.
- **Follow-up data:**
  - Postoperative delirium screening (4AT) assessed on day 3 (±1 day) following surgery.
  - Postoperative complications using the validated Comprehensive Complications Index (CCI) and complications as detailed below.
  - Date and destination of discharge from hospital .
  - Residential status and mobility at 120 days post-surgery.
  - Patient (or consultee reported) EQ5D5L at 120 days (±7) from surgery.
  - Days alive and at the original place of residence in the first 120 days (DAH120).
- **Clinical complications to be reported.**

The complications below align with the StEP-COMPAC group core outcomes for cardiovascular and pulmonary complications [1-5].

***Cardiovascular***

**Myocardial infarction**

Acute myocardial injury with clinical evidence of acute myocardial ischaemia and with detection of an increase or decrease in cardiac troponin (cTn) values with at least one value above the 99th percentile URL and at least one of the following:

(i) Symptoms of myocardial ischaemia;

(ii) New ischaemic ECG changes;

(iii) Development of pathological Q waves;

(iv) Imaging evidence of new loss of viable myocardium or new regional wall motion abnormality in a pattern consistent with an ischaemic aetiology;

(v) Identification of a coronary thrombus by angiography or autopsy;

Post-mortem demonstration of acute atherothrombosis in the artery supplying the infarcted myocardium.

Cardiac death in patients with symptoms suggestive of myocardial ischaemia and presumed new ischaemic ECG changes before cTn values become available.

**Cardiac death**

Death with a vascular cause and included those deaths after a myocardial infarction, cardiac arrest and cardiac revascularisation procedure.

**Pulmonary embolism**

Diagnosis of pulmonary embolism requires any one of the following:

(i) A high probability ventilation/perfusion lung scan;

(ii) An intraluminal filling defect of segmental or larger artery on a helical CT scan;

(iii) An intraluminal filling defect on pulmonary angiography;

(iv) A positive diagnostic test for deep venous thrombosis (positive compression ultrasound) and one of the following:

(a) Non-diagnostic (low or intermediate probability) ventilation/perfusion lung scan;

(b) Non-diagnostic (sub-segmental defects or technically inadequate study) helical CT scan.

**Deep venous thrombosis**

Diagnosis of deep venous thrombosis required any one of the following:

(i) A persistent intraluminal filling defect on contrast venography;

(ii) Non-compressibility of one or more venous segments on B-mode compression ultrasonography;

(iii) A clearly defined intraluminal filling defect on contrast enhanced CT.

**Atrial fibrillation**

New onset of irregularly irregular heart rate in the absence of P waves lasting at least 30 s or for the duration of the ECG recording (if <30 s).

**Postoperative pulmonary complications**

**Pneumonia**

Two or more serial chest radiographs with at least one of the following (one radiograph is sufficient for patients with no underlying pulmonary or cardiac disease):

(i) New or progressive and persistent infiltrates, (ii) consolidation, (iii) cavitation; AND at least one of the following:

(a) fever (>38^o^C) with no other recognised cause;

(b) leucopaenia (white cell count <4×10^9^.l^−1^) or leucocytosis (white cell count >12×10^9^.l^−1^);

(c) for adults >70 years old, altered mental status with no other recognised cause;

AND at least two of the following:

(a) new onset of purulent sputum or change in character of sputum or increased respiratory secretions or increased suctioning requirements;

(b) new onset or worsening cough or dyspnoea or tachypnoea;

(c) rales or bronchial breath sounds;

(d) worsening gas exchange (hypoxaemia, increased oxygen requirement, increased ventilator demand).

**Acute respiratory distress syndrome**

As per Berlin consensus criteria (2012):

a) Within one week of a known clinical insult or new worsening respiratory symptoms;

b) AND bilateral opacities on chest imaging, not fully explained by effusions, lobar/lung

collapse, or nodules;

c) AND respiratory failure not explained by cardiac failure or fluid overload (requires objective assessment, such as echocardiogram, to exclude hydrostatic oedema, if no risk factors are present);

d) AND supplemental oxygenation:

- Mild: PaO_2_:F_I_O_2_ 26.7-40.0 kPa with PEEP or CPAP ≥5 cmH_2_O;

- Moderate: PaO_2_:F_I_O2 13.3-26.6 kPa with PEEP ≥5 cmH_2_O;

- Severe: PaO_2_:F_I_O_2_ ≤13.3 kPa with PEEP ≥5 cmH_2_O.

As per StEP recommendations, the following will not be considered postoperative pulmonary complications: pulmonary embolism, pleural effusion, cardiogenic pulmonary oedema, pneumothorax and bronchospasm.

***Others***

**Fever**

Core body temperature >38.5°C more than 24 h following surgery with two readings within a 12-hour period.

**Clinical suspicion of infection and antibiotic use other than prophylaxis [6].**

Suspected site: Chest/Urinary/Blood/Wound/Other.

**Stroke**

Cerebral infarction or intracerebral haemorrhage on CT or MRI, or new neurological signs (paralysis, weakness, or speech difficulties) lasting >24 h or leading to earlier death.

**Acute Kidney Injury Stage 3**

According to the KIDGO consensus definition of acute kidney injury (2012): serum creatinine 3 times baseline OR ≥4 mg.dl^-1^ (≥ 353.6 mmol.l^-1^) increase OR initiation of renal replacement therapy AND/OR urine output <0.3 ml.kg^-1^.h^-1^ for ≥24 h OR no urine output ≥12 h. In many cases, this will be visible as an alert in the electronic blood results.

*Note- standard care patients may receive an arterial line if the treating clinician(s) feel this becomes indicated during the care of the patient. As far as possible data fields have been aligned with those in the National Hip Fracture Database and core outcome sets for perioperative and hip fracture research [7, 8].

1. Abbott T, Fowler A, Pelosi P, et al. A systematic review and consensus definitions for standardised end-points in perioperative medicine: pulmonary complications. *Br J Anaesth* 2018; **120**: 1066-79.

2. Beattie WS, Lalu M, Bocock M, et al. Systematic review and consensus definitions for the Standardized Endpoints in Perioperative Medicine (StEP) initiative: cardiovascular outcomes. *Br J Anaesth* 2021; **126**: 56-66.

3. McIlroy D, Bellomo R, Billings IV, et al. Systematic review and consensus definitions for the Standardised Endpoints in Perioperative Medicine (StEP) initiative: renal endpoints. *Br J Anaesth* 2018; **121**: 1013-24.

4. Barnes J, Hunter J, Harris S, et al. Systematic review and consensus definitions for the Standardised Endpoints in Perioperative Medicine (StEP) initiative: infection and sepsis. *Br J Anaesth* 2019; **122**: 500-8.

5. Haller G, Bampoe S, Cook T, et al. Systematic review and consensus definitions for the Standardised Endpoints in Perioperative Medicine initiative: clinical indicators. *Br J Anaesth* 2019; **123**: 228-37.

6. Yeung J, Jhanji S, Braun J, et al. Volatile vs Total intravenous Anaesthesia for major non-cardiac surgery: a pragmatic randomised triaL (VITAL). *Trials* 2024; **25**: 414.

7. Boney O, Moonesinghe SR, Myles PS, et al. Core Outcome Measures for Perioperative and Anaesthetic Care (COMPAC): a modified Delphi process to develop a core outcome set for trials in perioperative care and anaesthesia. *Br J Anaesth* 2022; **128**: 174-85.

8. Haywood KL, Griffin XL, Achten J, Costa ML. Developing a core outcome set for hip fracture trials. *Bone Joint J* 2014; **96**: 1016-23.
