# Supplementary material for: Continuous ARterial monitoring in Elderly and Frail patients for hip fractUre surgery to prevent Low blood pressure – the CAREFUL Study Protocol
Source: Anaesth Rep. 2026 Apr 9;14(1):e70059. doi: 10.1002/anr3.70059 (PMC13062759; doi:10.1002/anr3.70059)
Supplement: Supplementary file 3 — Supporting Information 3. Adverse and serious adverse events. [file ANR3-14-e70059-s005.docx]

**Supporting information 3: Adverse and serious adverse events**

| Adverse Event (AE) | Any untoward medical occurrence in a study participant taking part in the study, which does not necessarily have to have a causal relationship with the intervention.    An AE can be any unfavourable/unintended sign, symptom/disease temporarily associated with the intervention. |
| --- | --- |
| Serious Adverse Event (SAE) | An AE that:   - led to death; - led to serious deterioration in the health of the participant; - resulted in a permanent impairment of a body structure/function; - required inpatient hospitalisation/prolongation of existing hospitalisation; - resulted in medical/surgical intervention to prevent permanent impairment to a body structure/function; - resulted in a life-threatening illness/injury.     The term “*life-threatening*” in the definition of “*serious*” refers to an event in which the study participant was at risk of death at the time of the event; it does not refer to an event which hypothetically might have caused death if it were more severe.    Other events that may not result in death, are not life-threatening or do not require hospitalisation, may be considered an SAE when, based upon appropriate medical judgement, the event may jeopardise the study participant and may require medical/surgical intervention to prevent one of the outcomes listed above.  The causal relationship with the study intervention will be assessed for any SAEs reported. |
| Unexpected AE/SAE | Any AE/SAE on health, safety, any life-threatening problem or death caused by, or associated with the intervention, if that effect, problem, or death was not previously identified in nature, severity or degree of incidence in the study protocol, or any other unanticipated serious problem associated with the intervention that related to the rights, safety or welfare of the study participant. |

**Reporting of adverse events**

All AEs/SAEs occurring during the study period, as observed by the CI/PI, research team, clinical team, or participant, will be recorded. All AEs requiring reporting will be recorded on an eCRF.

The following information will be recorded:

- description;
- date of onset and end date;
- severity and relatedness to the intervention;
- action taken.

Follow-up information should be provided as necessary.

The relationship of AEs/SAEs to the study intervention will be assessed by the Sponsor/CI. Serious adverse events will be monitored until they are resolved or up to 30 days after study end for the participant.

The CAREFUL Study is a non-CTIMP study, and invasive continuous arterial blood pressure monitoring may already be used in some patients in this group as part of routine clinical care. Many expected postoperative complications, which are of importance, will be collected as study outcomes (need for blood transfusion, death) and will not be reported as separate AEs where they are already captured. This approach is in line with other interventional studies in peri-operative medicine where high rates of AEs are expected within the normal population of interest [1].

Some AEs/SAEs may occur as a result of the study intervention, including in the standard care group, where an arterial line may be inserted for clinical reasons during patient care. Where any of these have occurred after the patient has been enrolled into the study, they do not need to be reported immediately. The expected AE/SAE should be recorded in the appropriate section of the eCRF. The potential events related to the study interventions include:

- Vascular injury resulting in compromise to the blood supply of the hand following arterial cannulation;
- Need for any surgical opinion related to complications associated with the insertion of an arterial line;
- Retained guidewire or catheter following arterial cannulation;
- Infection at the site of arterial access requiring antibiotic treatment;
- Transfer of a patient to a clinical area with an arterial line, which does not usually care for patients with arterial lines;
- Any other event that the local PI or research delivery team deems to meet the criteria for an AE and is related to the intervention, which is not otherwise captured as an outcome of the study.

**Unexpected adverse events**

For the purpose of safety reporting of this study, only unexpected SAEs potentially related to the intervention will be reported immediately to the study team.

When the local research team becomes aware of an unexpected SAE in a study participant, the local PI will review the SAE and consider the association of the event with the intervention. If the PI assesses the SAE to be potentially related, the details of the event will be entered on a SAE reporting form on the database (see Section 10.2). The research team will notify the study team via email **within 24 hours** of the PI becoming aware of the event.

Once received, causality and expectedness will be confirmed by the CI or nominated deputy. SAEs that are deemed unexpected and related to the study will be notified to the REC **within 15 days**. All such events will also be reported to the Trial Steering Committee (TSC) at their next meetings.

1. Yeung J, Jhanji S, Braun J, et al. Volatile vs Total intravenous Anaesthesia for major non-cardiac surgery: a pragmatic randomised triaL (VITAL). *Trials* 2024; **25**: 414.
