# Supplementary material for: Continuous ARterial monitoring in Elderly and Frail patients for hip fractUre surgery to prevent Low blood pressure – the CAREFUL Study Protocol
Source: Anaesth Rep. 2026 Apr 9;14(1):e70059. doi: 10.1002/anr3.70059 (PMC13062759; doi:10.1002/anr3.70059)
Supplement: Supplementary file 4 — Supporting Information 4. Consent details. [file ANR3-14-e70059-s006.docx]

**Supporting information 4: Consent details**

**Layer 1: Patient consent**

- Assume that the patient has capacity, unless there is evidence to suggest otherwise. Consult the clinical team, if needed;
- If the patient can consent, they must be approached first;
- Consent can be verbal if witnessed;
- Patients will be presented with the participant information sheet and a one-page infographic summary. The research team will summarise these and answer any questions the patient may have about the study.
- If the patient does not have the capacity to consent, then a personal consultee can be approached (layer 2).

**Layer 2: Personal consultee approach**

- A personal consultee (next of kin, close family member, friend or carer) may provide agreement for a patient to participate. This is in line with section 32 subsection 9b of the Mental Capacity Act 2005.
- If the patient is not expected to regain capacity at any point in the future, then a consultee agreement can suffice for the entire study.
- If the patient is expected to regain capacity they should be approached at a point later in the study to confirm consent.
- Personal consultee agreement can be over the phone. This can be verbal or an electronic link can be emailed to the consultee to complete.
- If the patient lacks capacity and personal consultees cannot be contacted, then a clinical consultee can be used (layer 3).

**Layer 3: Clinical consultee approach**

- If the patient lacks capacity and no personal consultee is contactable, then a clinical consultee can be approached. There will be a non-treating doctor who is neither the CI, PI, nor on the local delegation log. They can be an anaesthetist, orthopaedic surgeon or orthogeriatrician.
- Further consent should be sought from the patient or a personal consultee following surgery, if this is possible. If this is not possible, a clinical consultee agreement can suffice for the duration of the study.

If either clinical or personal consultees are used and the patient later has capacity and refuses to consent to continue in the study, then their involvement in the study will end at that point. In this case, data collected up to this point will be used anonymously for study analysis, unless a patient requests its removal. We will give the patient the option to allow us to continue collecting hospital and other outcome data without contact for patient-reported outcome measures, if this is acceptable to them. The above patient and consultee approach methodology is consistent with other major studies in this area [1].

A copy of the consent form will be provided to the patient/consultee and recorded in the patient’s medical notes.

Consent will include all aspects of this protocol and future use of the data (through linkage to other data bases, including, but not limited to, the National Hip Fracture Database and Hospital Episode Statistics).

1. Costa ML, Griffin XL, Achten J, et al. World Hip Trauma Evaluation (WHiTE): framework for embedded comprehensive cohort studies. *BMJ Open* 2016; **6**: e011679.
