# Supplementary material for: Continuous ARterial monitoring in Elderly and Frail patients for hip fractUre surgery to prevent Low blood pressure – the CAREFUL Study Protocol
Source: Anaesth Rep. 2026 Apr 9;14(1):e70059. doi: 10.1002/anr3.70059 (PMC13062759; doi:10.1002/anr3.70059)
Supplement: Supplementary file 5 — Supporting Information 5. Data management and storage. [file ANR3-14-e70059-s002.docx]

**Supporting information 5: Data management and storage**

The study data and electronic case report forms will be collected and managed using REDCap, hosted by South Tees Hospitals NHS Foundation Trust. REDCap is a secure, web-based software platform designed to support data capture for research studies, providing an intuitive interface for validated data capture; audit trails for tracking data manipulation and export procedures; automated export procedures for seamless data downloads to common statistical packages; and procedures for data integration and interoperability with external sources.

We will comply with all national legislation and Sponsor requirements to protect patient data and confidentiality. Where appropriate data will be minimised to the minimum necessary at all points of transfer to prevent inadvertent loss of data. Data will be retained for up to 5 years following either the completion of this project or any subsequent study in which data from this project is linked with other data sources.
