# Supplementary material for: Continuous ARterial monitoring in Elderly and Frail patients for hip fractUre surgery to prevent Low blood pressure – the CAREFUL Study Protocol
Source: Anaesth Rep. 2026 Apr 9;14(1):e70059. doi: 10.1002/anr3.70059 (PMC13062759; doi:10.1002/anr3.70059)
Supplement: Supplementary file 6 — Supporting Information 6. Study monitoring and sponsorship. [file ANR3-14-e70059-s001.docx]

**Supporting information 6: Study monitoring and sponsorship**

**Trial coordination**

The trial coordination will be managed by the South Tees Academic Centre for Surgery (ACeS) in collaboration with the Sponsor and the chief investigator. The ACeS team will include the trial manager, trial coordinators, data management and support staff.

**Trial management group**

The trial management group will monitor the day-to-day management of the study. There will be regular meetings via teleconferencing software, supplemented by face-to-face meetings approximately monthly during the study. Meetings will be chaired by the CI or their nominated deputy and will include all co-applicants, sponsor representatives, and, where necessary, members of the study management team from ACeS, as well as other research staff, medically qualified trainees, allied health professionals or students working on the project. All members will be up to date with local information governance training.

**Trial steering committee**

Independent oversight of the study will be conducted by the trial steering committee (TSC), which will provide overall supervision of The CAREFUL Study on behalf of the Sponsor and Funder. They will ensure that the project is conducted to the rigorous standards set out in the UK Policy Framework for Health and Social Care Research and the Guidelines for Good Clinical Practice (GCP).

The TSC will monitor the progress of the study and provide independent advice. The TSC will consist of the chief and co-chief investigator, a delegated member of the trial management team from the Academic Centre for Surgery and an external clinical representative and methodological expert. A Sponsor representative will be invited to attend the TSC meetings. Other co-applicants or collaborators may also be invited to attend, with the agreement of the Chair. The TSC will meet every six months over the 30-month study period.

**Inspection of records**

Study-related monitoring on behalf of the Sponsor and regulatory inspection(s) will be permitted. In the event of monitoring/regulatory inspection, the CI/PI will agree to allow Sponsor/regulatory inspection representatives direct access to all study records and source documentation.

**Study monitoring**

A research project manager from the study management team will visit the Investigator site before the start of the study, per the local monitoring plan and risk assessment (if required). This visit may be remote. As this study has been evaluated as low risk by the study Sponsor’s risk assessment, central and remote monitoring will be utilised. Data will be reviewed for compliance with the study protocol and accuracy in relation to source documents. Following written standard operating procedures (SOPs), it will be verified that the study is conducted, and data generated, documented and reported in compliance with the study protocol, the local monitoring plan and the applicable regulatory requirements.

**Activities performed by Sponsor representatives**

The Sponsor will be responsible for performing the following activities:

- Auditing the study as required according to sponsor monitoring procedures and ensuring any exceptions are justified and documented;
- Training of the CIs in the Sponsor’s procedure for reporting serious breaches of GCP.
